# Atropine-induced intraocular pressure changes and anterior segment structural parameters in dogs assessed by ultrasound biomicroscopy

**DOI:** 10.3389/fvets.2026.1820977

**Published:** 2026-05-20

**Authors:** Mirae Lee, Donghee Kim, Ji Seung Jung, Jiyi Hwang, Haemi Seol, Sooyeon Lee, Kyung-Mee Park

**Affiliations:** Laboratory of Veterinary Surgery and Ophthalmology, College of Veterinary Medicine, Chungbuk National University, Cheongju, Republic of Korea

**Keywords:** aqueous humor outflow, atropine-induced mydriasis, ciliary body motion, ciliary cleft morphology, intraocular pressure changes, ultrasound biomicroscopy

## Abstract

**Introduction:**

In dogs, intraocular pressure (IOP) responses to topical atropine administration vary, and the anatomical basis for this variability remains unclear.

**Materials and methods:**

Using ultrasound biomicroscopy (UBM), anterior segment morphology was assessed in 52 eyes from 27 clinically normal dogs before and 20 minutes after atropine administration (post-AT).

**Results:**

The mean change in IOP was small, but responses varied across eyes. Approximately 29% showed increases considered clinically relevant. Eyes were categorized into a high group (≥4 mmHg increase) and a stable group (<4 mmHg change) according to post-AT IOP change. Ciliary cleft area (CCA) increased significantly in the stable group and was accompanied by posterior-outward displacement of the ciliary body. In the high group, CCA tended to decrease, with relative anterior displacement of the ciliary body, although these changes were not consistently significant across parameters. In contrast, traditional anterior chamber angle parameters did not differ between groups.

**Discussion:**

Heterogeneous post-AT IOP responses appear to be associated with group-dependent differences in ciliary body configuration and ciliary cleft morphology. Variation in these structures may reflect underlying anatomical factors associated with IOP variability. UBM-based assessment may help interpretation of atropine-induced IOP changes.

## 1 Introduction

Pharmacologic pupil dilation can influence intraocular pressure (IOP) in both humans and animals, a finding supported by previous clinical and experimental studies. IOP responses vary across species and pharmacologic agents, and individual anatomical characteristics may influence their clinical implications (1–4). Most eyes show minimal IOP change after dilation, but some eyes show greater pressure elevation, which may reflect underlying structural factors. Examining these anatomical features may help clarify the basis of pronounced IOP elevation in certain eyes (5–8).

IOP depends on the balance between aqueous humor production and resistance to outflow within the anterior segment (9). Aqueous humor exits the eye through two main pathways, the trabecular and uveoscleral routes. In dogs, the ciliary cleft (CC) serves as a shared outflow-related anatomical region (10, 11). It is closely associated with trabecular structures and contiguous with the ciliary body and supraciliary region involved in uveoscleral drainage (10, 11). Structural variation in these regions may affect aqueous humor drainage and IOP stability (12). Ultrasound biomicroscopy (UBM) enables noninvasive, high-resolution imaging of deep anterior segment structures and assessment of morphological changes after pharmacologic treatment (12–14).

In veterinary and experimental ophthalmology, pharmacologic dilation is commonly achieved with atropine. This agent acts as a muscarinic receptor antagonist at the level of the iris sphincter and ciliary muscle, resulting in sustained mydriasis and cycloplegia (15, 16). Beyond its mydriatic action, atropine is used clinically for pain control, uveitis treatment, and prevention of posterior synechiae (17). The literature does not present a uniform pattern regarding IOP response. While canine and adult human studies report IOP elevation, pediatric and equine investigations describe minimal or decreased values (18–21). Studies in human populations report clinically significant IOP elevation after pharmacologic dilation in glaucomatous and high-risk eyes, with comparatively small average changes in non-glaucomatous groups (22, 23). Such patterns suggest that anatomical configuration may influence atropine-associated IOP behavior.

This study employed UBM to characterize anterior segment morphological changes following topical 1% atropine in clinically normal dogs. Particular emphasis was placed on the dynamic relationship between the ciliary body and the CC, as well as its association with inter-individual differences in IOP response following topical atropine administration (post-AT).

## 2 Materials and methods

### 2.1 Animals and clinical examination

Between October 2024 and March 2025, 52 eyes from 27 dogs were prospectively recruited at the Veterinary Teaching Hospital of Chungbuk National University (Cheongju, Republic of Korea) for an interventional study. Enrollment was limited to eyes without clinically detectable ocular abnormalities. Written informed consent was obtained from all owners, and the study protocol was approved by the Chungbuk National University Institutional Animal Care and Use Committee (IACUC; CBNUA-24-0095-02).

All dogs received a thorough ophthalmic examination, including Schirmer tear test (Gulden Ophthalmics), rebound tonometry (TonoVet Plus®, iCare), menace response, pupillary light and dazzle reflex evaluation, slit-lamp biomicroscopy (MW50D, SHIGIYA), gonioscopy using a Koeppe lens (Ocular Instruments Inc.), indirect ophthalmoscopy (Pan Retinal® 2.2, Volk Optical), and ultrasound biomicroscopy (UBM; VuPAD®, Sonomed Escalon).

### 2.2 IOP measurement and UBM imaging

IOP and UBM measurements were obtained before atropine administration (pre-AT) and 20 min post-AT (Atropine Sulfate 1%®; Alcon, Fort Worth, TX) (20, 24). All examinations were performed around 10:00 a.m. by a single examiner under standardized conditions to minimize diurnal variation (25). Examinations were conducted in a calm environment to minimize stress.

At baseline, IOP was measured three times in each eye, and the average value was used for analysis. Topical anesthesia was then applied, followed by UBM imaging. UBM images were acquired in the dorsal quadrant with the probe positioned perpendicularly to the corneoscleral limbus, avoiding pressure on the globe. Three images per eye were averaged for analysis, and the same protocol was repeated at post-AT.

**Figure 1:**
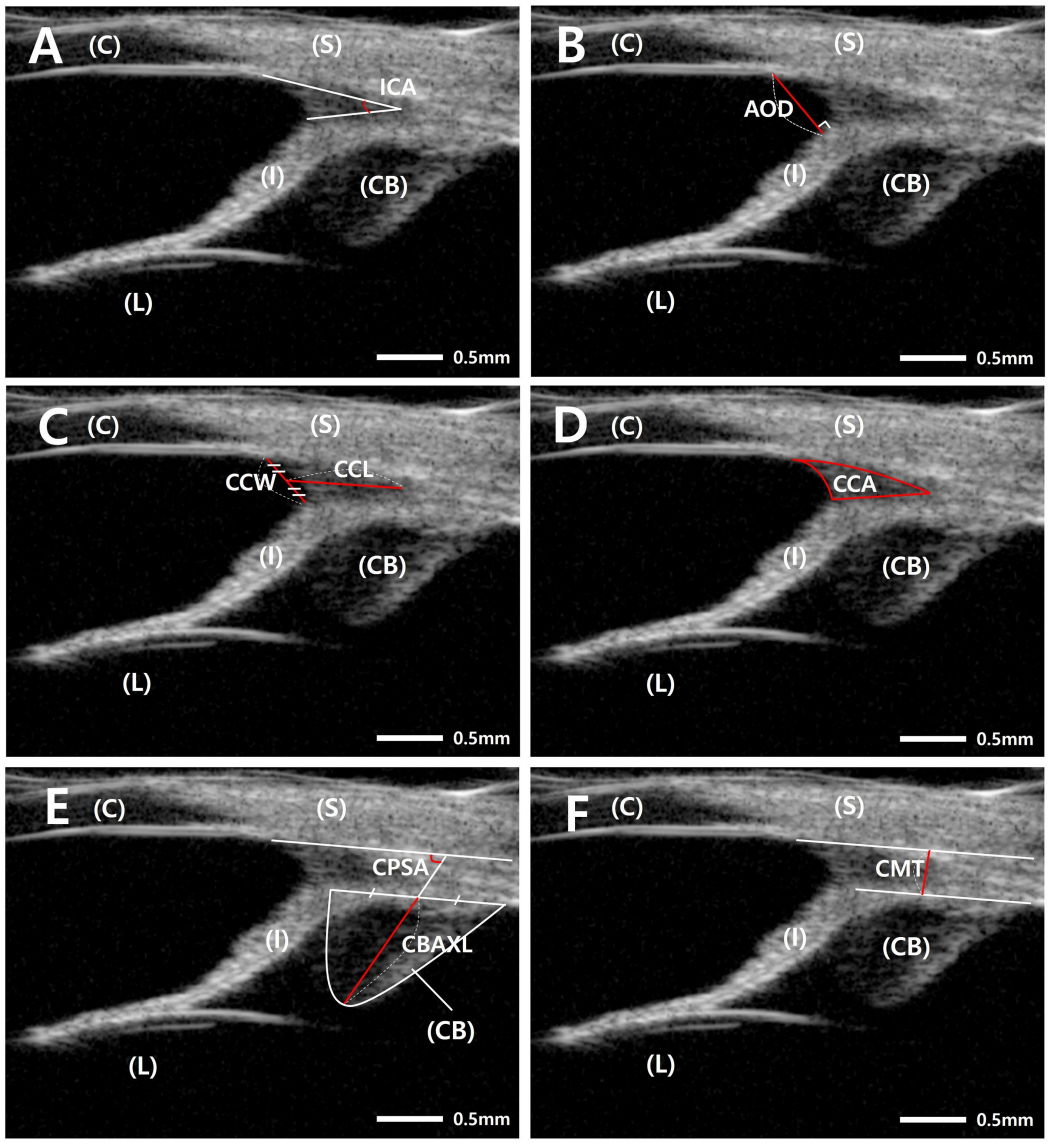
Measurement of anterior segment parameters using ultrasound biomicroscopy in dogs. **(A)** The iridocorneal angle (ICA) was measured as the angle formed between the inner corneoscleral wall and the iris root. **(B)** Angle-opening distance (AOD) was defined as the perpendicular linear distance from the endpoint of Descemet’s membrane to the anterior iris surface. **(C)** Ciliary cleft width (CCW) was defined as the linear distance from the intersection of the outer margin of the pectinate ligament and the inner scleral boundary to the anterior surface of the iris root. Ciliary cleft length (CCL) was defined as the distance from the angle recess to the central point of the CCW. **(D)** Ciliary cleft area (CCA) was defined as the area bounded by the inner scleral wall, the iris root surface, and the CCW boundary. **(E)** Ciliary body axial length (CBAXL) was defined as the distance from the apex of the dome-shaped ciliary body to the midpoint of its base. The ciliary process–sclera angle (CPSA) was defined as the angle formed between the longitudinal axis of the ciliary body and the adjacent inner scleral wall. **(F)** The ciliary muscle thickness (CMT) was measured as the perpendicular distance from the inner scleral boundary to the outer margin of the ciliary body. Scale bars = 0.5 mm. CB, ciliary body; C, cornea; I, iris; L, lens; S, sclera.

Anterior segment parameters were measured using the built-in UBM caliper software, with definitions shown in Figure 1 (6, 26–32)

ICA was determined as the angle formed between the inner scleral wall and the iris root (Figure 1A). Angle-opening distance (AOD) was measured as the perpendicular distance from Descemet’s membrane termination to the anterior iris surface (Figure 1B).

CC morphology was characterized using CC width (CCW), measured from the intersection of the outer margin of the pectinate ligament and the inner scleral boundary to the anterior iris surface; CC length (CCL), measured from the angle recess to the central region of the CCW; and CC area (CCA), calculated as the area enclosed by the inner scleral wall, iris root surface, and CCW boundary (Figures 1C,D).

Ciliary body parameters included ciliary body axial length (CBAXL), defined as the distance from the apex of the dome-shaped ciliary body to the midpoint of its base, the ciliary process-sclera angle (CPSA), and ciliary muscle thickness (CMT), measured as the perpendicular distance from the inner scleral boundary to the outer margin of the ciliary body (Figures 1E,F).

### 2.3 Experimental groups

Eyes were classified according to post-AT IOP response. An increase in IOP of ≥4 mmHg from baseline was used to define the high group, whereas eyes with changes of <4 mmHg were classified as the stable group.

This threshold was selected as a clinically oriented exploratory cutoff to identify IOP elevations exceeding the reported physiologic diurnal fluctuation range in dogs (25). Importantly, this threshold was pre-specified during study design based on prior literature and clinical considerations. In addition, the same cutoff has been used in a previous UBM-based study evaluating mydriatic-induced IOP changes (0.5% tropicamide-0.5% phenylephrine), allowing direct comparison across pharmacologic mydriatic models (33).

Accordingly, this classification is best interpreted as a clinically oriented and exploratory framework rather than a universally validated responder definition. All eyes included had baseline IOP values within the clinically normal range.

### 2.4 Statistical analysis

Statistical analyses were conducted using SPSS software (version 17.0; SPSS Inc.) and GraphPad Prism (version 11; GraphPad Software). Data distribution was assessed prior to analysis. Differences in sex and breed distribution between groups were evaluated with the chi-square test, whereas age and body weight were compared using the Mann–Whitney U test.

To address correlations arising from bilateral eyes, IOP and UBM parameters were analyzed using linear mixed-effects models (LMMs). Group (high vs. stable) and time (pre-AT vs. post-AT) were modeled as fixed effects, while dog identity was specified as a random effect. Eyes were categorized into stable and high groups based on the change in IOP (*Δ*IOP) at post AT. Changes in parameters (Δ) were calculated as post-atropine (post-AT) minus pre-atropine (pre-AT) values. Baseline comparisons between groups were performed using Welch’s t-test.

Correlation analyses were performed to evaluate the relationships between changes in anterior segment parameters and ΔIOP. Spearman rank correlation coefficients (r) were calculated to assess monotonic relationships between variables. Correlations were assessed across all eyes, and additional subgroup analyses were performed within the stable and high groups. Statistical significance was set at *p* < 0.05.

## 3 Results

### 3.1 Canine demographics

The stable group included 21 dogs (37 eyes), and the high group consisted of 10 dogs (15 eyes). The mean age was 6.30 ± 2.50 years in the stable group and 4.64 ± 3.85 years in the high group. The mean body weight was 5.36 ± 2.26 kg and 4.78 ± 1.72 kg in the respective groups.

The stable group included 10 castrated males, 7 spayed females, 3 intact females, and 1 intact male (dogs). The high group included 5 castrated males, 3 spayed females, and 1 intact male (dogs).

Regarding breed distribution, the stable group included Mixed (*n* = 12), Pomeranian (*n* = 10), Maltese (*n* = 3), Miniature Poodle (*n* = 3), Shih Tzu (*n* = 3), Bichon Frise (*n* = 2), Yorkshire Terrier (*n* = 2), and Dachshund (*n* = 2). The high group included Mixed (*n* = 3), Miniature Poodle (*n* = 3), Shih Tzu (*n* = 3), Maltese (*n* = 2), Pomeranian (*n* = 2), and Bichon Frise (*n* = 2). Unless otherwise specified, values of *n* refer to the number of eyes.

Of the 27 dogs, 25 contributed both eyes, including four dogs with one eye assigned to each group. Two dogs contributed only one eye due to contralateral enucleation or corneal ulceration. No significant differences were observed between the stable and high groups with respect to age (*p* = 0.49), body weight (*p* = 0.33), sex distribution (*p* = 0.61), or breed distribution (*p* = 0.70), indicating comparable baseline demographics (Table 1).

**Table 1:** Demographic characteristics and group allocation of the enrolled dogs.

Dog	Gender	Weight	Age (years)	Breed	OD	OS
1	SF	7.67	4	Mixed	Stable	Stable
2	CM	4.90	6	Miniature Poodle	High	High
3	SF	3.60	3	Pomeranian	High	High
4	CM	8.46	8	Mixed	Stable	Stable
5	SF	2.82	8	Miniature Poodle	Stable	Stable
6	CM	7.49	6	Shih Tzu	Stable	Stable
7	IF	5.09	4	Mixed	–	Stable
8	IF	5.04	4	Mixed	Stable	Stable
9	IM	2.70	11	Yorkshire Terrier	Stable	Stable
10	CM	4.30	1	Bichon Frise	High	High
11	CM	6.24	4	Bichon Frise	Stable	Stable
12	SF	2.00	6	Pomeranian	Stable	Stable
13	SF	1.31	3	Pomeranian	Stable	Stable
14	CM	8.43	5	Mixed	Stable	Stable
15	CM	3.90	7	Maltese	Stable	Stable
16	SF	4.60	9	Mixed	Stable	Stable
17	CM	7.70	8	Pomeranian	Stable	Stable
18	IM	3.63	6	Maltese	–	High
19	CM	5.83	5	Pomeranian	Stable	Stable
20	CM	4.95	5	Pomeranian	Stable	Stable
21	CM	2.11	6	Maltese	High	Stable
22	CM	8.18	5	Miniature Poodle	Stable	High
23	SF	4.64	1	Mixed	High	Stable
24	SF	7.43	11	Shih Tzu	Stable	High
25	CM	6.70	9	Shih Tzu	High	High
26	IF	6.30	10	Dachshund	Stable	Stable
27	SF	3.38	1	Mixed	High	High

Dogs were assigned to the stable group (<4 mmHg IOP change) or the high group (≥4 mmHg IOP elevation) based on intraocular pressure response after 1% atropine administration. CM, castrated male; IF, intact female; IM, intact male; OD, right eye; OS, left eye; SF, spayed female.

### 3.2 Changes in IOP at pre-AT and post-AT in all eyes

**Figure 2:**
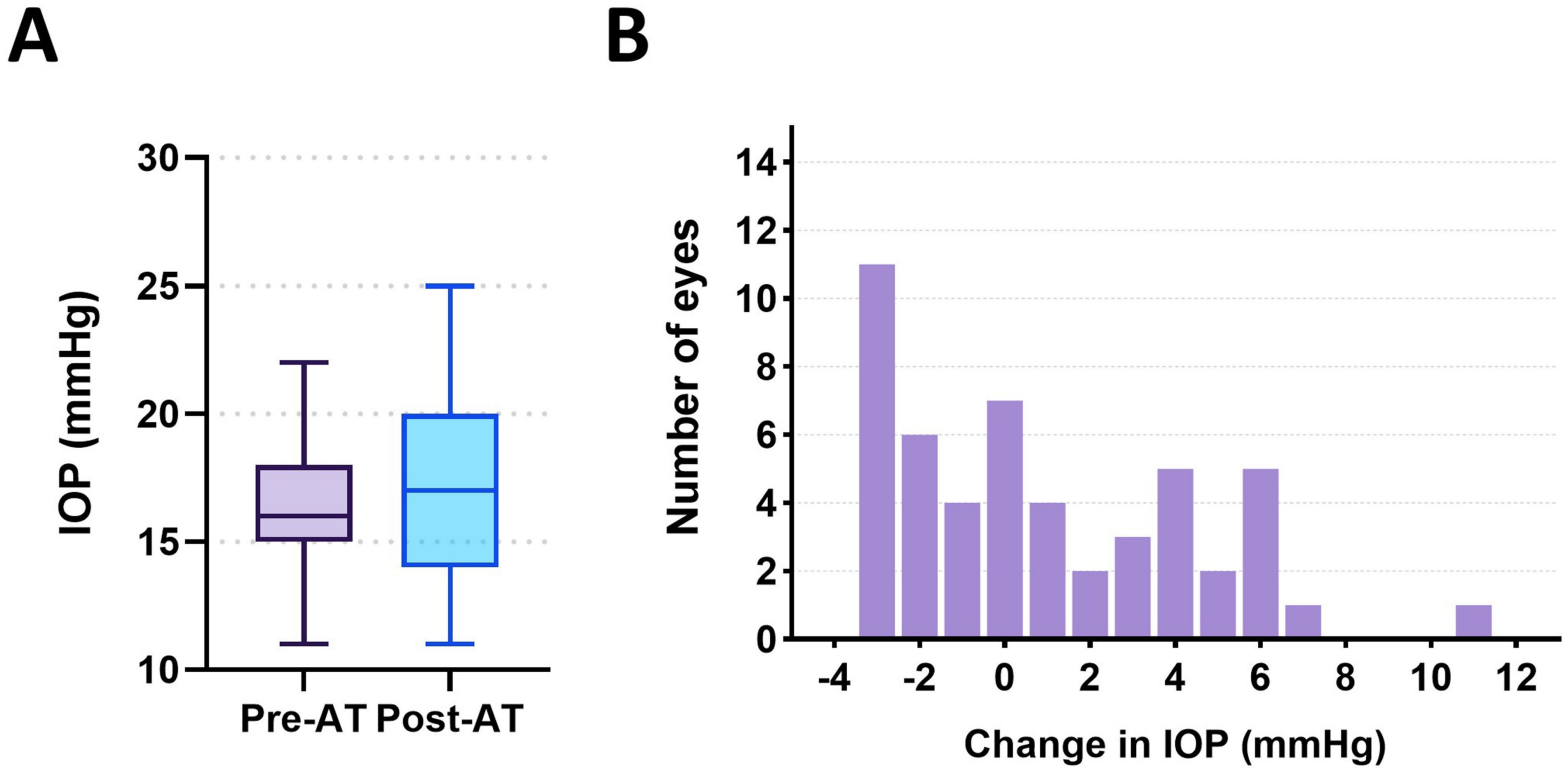
Change in intraocular pressure (IOP) after 1% atropine. **(A)** Boxplots comparing IOP measured before (pre-AT) and after topical administration of 1% atropine (post-AT). **(B)** Frequency distribution of IOP changes (post–pre) across all examined eyes.

Changes in IOP across all eyes were first evaluated to assess the overall response to atropine (Figure 2). The mean IOP at pre-AT was 16.40 ± 2.90 mmHg. At post-AT, the mean IOP increased to 17.30 ± 3.30 mmHg, with a mean change of +0.90 ± 3.50 mmHg (Figure 2A). At no time point did IOP exceed 25 mmHg in any of the examined eyes, a threshold generally regarded as the upper limit of the normal range when measured using rebound tonometry (TonoVet Plus®).

IOP changes varied across eyes, with both decreases and increases observed (Figure 2B). IOP reductions of −1.00 to −3.00 mmHg were commonly observed, whereas larger increases occurred less often. In a subset of eyes, IOP increases of ≥4.00 mmHg were observed, with a maximum increase of 11.00 mmHg.

### 3.3 IOP in stable and high groups

**Figure 3:**
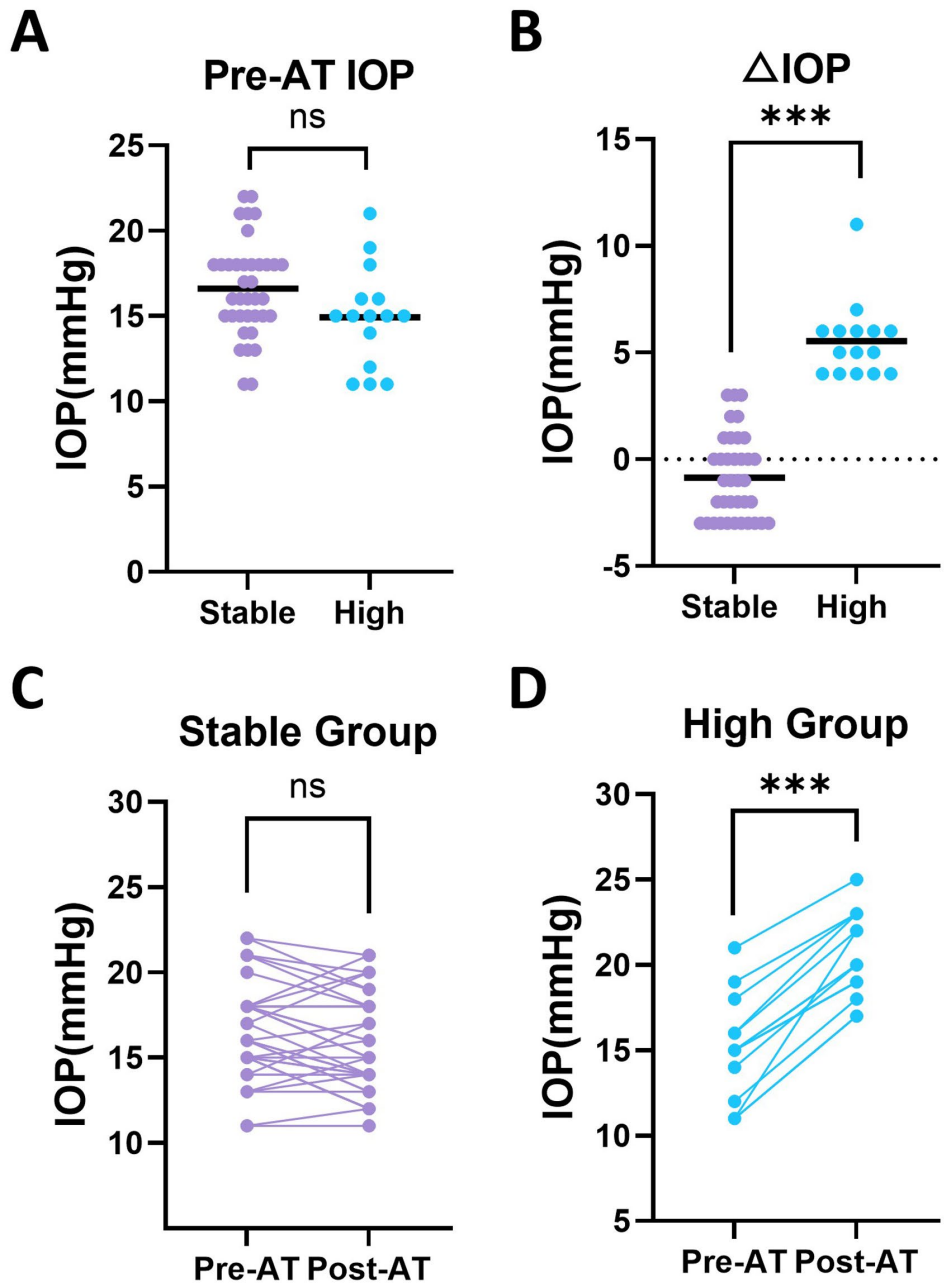
Baseline and post-atropine (post-AT) intraocular pressure (IOP) changes in stable and high groups. **(A)** Baseline IOP measured before atropine administration (pre-AT) in the stable and high groups. **(B)** Change in IOP (ΔIOP; post-AT minus pre-AT) in the stable and high groups. **(C)** Paired baseline and post-AT IOP values in the stable group. **(D)** Paired baseline and post-AT IOP values in the high group. Statistical analyses were performed using linear mixed-effects models. Each dot represents an individual eye. Horizontal bars indicate mean values in panels A and B. Asterisks indicate statistically significant differences. Asterisks above paired data represent within-group comparisons between pre-AT and post-AT values, whereas asterisks above grouped data represent between-group comparisons. *p < 0.05, **p < 0.01, ***p < 0.001; ns, not significant.

Eyes were categorized into stable and high groups based on ΔIOP, and differences between the groups were analyzed (Figure 3). LMM analysis identified a significant group × atropine interaction effect on IOP (*p* < 0.001), indicating that the effect of atropine on IOP differed between the two groups (Table 2). Baseline IOP values were comparable between the stable and high groups (Figure 3A). At post-AT, IOP did not change significantly in the stable group (16.62 ± 2.81 vs. 15.76 ± 2.81 mmHg, *p* = 0.189), whereas a significant increase was observed in the high group (14.93 ± 2.69 vs. 20.47 ± 2.69 mmHg, *p* < 0.001) (Table 2). The corresponding mean changes were −0.86 ± 1.96 mmHg in the stable group and +5.53 ± 1.81 mmHg in the high group. These contrasting changes are summarized in Figure 3B and further visualized in paired baseline and post-AT plots for each group (Figures 3C,D).

**Table 2:** Changes in intraocular pressure and anterior segment parameters following topical 1% atropine administration, as assessed by linear mixed-effects models (LMM).

Parameter	Stable group (*n* = 37 eyes)			High group (*n* = 15 eyes)			Atropine (*p*)	Group × atropine interaction (*p*)
	Pre-AT	Post-AT	Within-group (*p*)	Pre-AT	Post-AT	Within-group (*p*)		
IOP (mmHg)	16.62 ± 2.81	15.76 ± 2.81	0.189	14.93 ± 2.69	20.47 ± 2.69	**<0.001**	**<0.001**	**<0.001**
ICA (°)	22.32 ± 7.41	17.87 ± 7.41	**0.012**	25.37 ± 8.39	20.60 ± 8.39	0.131	**0.007**	0.926
AOD (mm)	0.85 ± 0.17	0.94 ± 0.17	**0.025**	0.93 ± 0.24	0.98 ± 0.24	0.558	0.089	0.633
CCW (mm)	0.67 ± 0.16	0.71 ± 0.16	0.338	0.71 ± 0.14	0.70 ± 0.14	0.817	0.725	0.477
CCL (mm)	1.18 ± 0.26	1.24 ± 0.26	0.293	1.04 ± 0.20	1.08 ± 0.20	0.630	0.347	0.799
CCA (mm^2^)	0.27 ± 0.07	0.32 ± 0.07	**0.002**	0.28 ± 0.07	0.24 ± 0.07	0.084	0.733	**0.002**
CBAXL (mm)	1.95 ± 0.30	1.73 ± 0.30	**0.003**	1.81 ± 0.19	1.96 ± 0.19	**0.033**	0.623	**0.002**
CPSA (°)	44.39 ± 6.69	48.58 ± 6.69	**0.009**	49.37 ± 6.35	47.53 ± 6.35	0.434	0.415	**0.038**
CMT (mm)	0.63 ± 0.09	0.71 ± 0.09	**<0.001**	0.73 ± 0.10	0.68 ± 0.10	0.177	0.483	**0.001**

Values are presented as mean ± standard deviation (SD). Standard deviations were derived from standard errors estimated by LMM using the formula SD=SE×√ *n*. Within-group (p) indicates pre- vs post-AT comparisons within each group. Atropine (p) represents the overall effect of atropine, and group × atropine interaction (p) indicates differential effects between groups, as assessed by LMM accounting for within-dog correlation between eyes. Significant *p*-values (*p* < 0.05) are shown in bold.

### 3.4 Anterior segment changes following topical atropine administration

Anterior segment parameters were evaluated pre-AT and post-AT to characterize atropine-induced structural changes assessed by UBM. Group-dependent changes in IOP and anterior segment parameters at post-AT are summarized in Table 2.

#### 3.4.1 Anterior chamber changes

**Figure 4:**
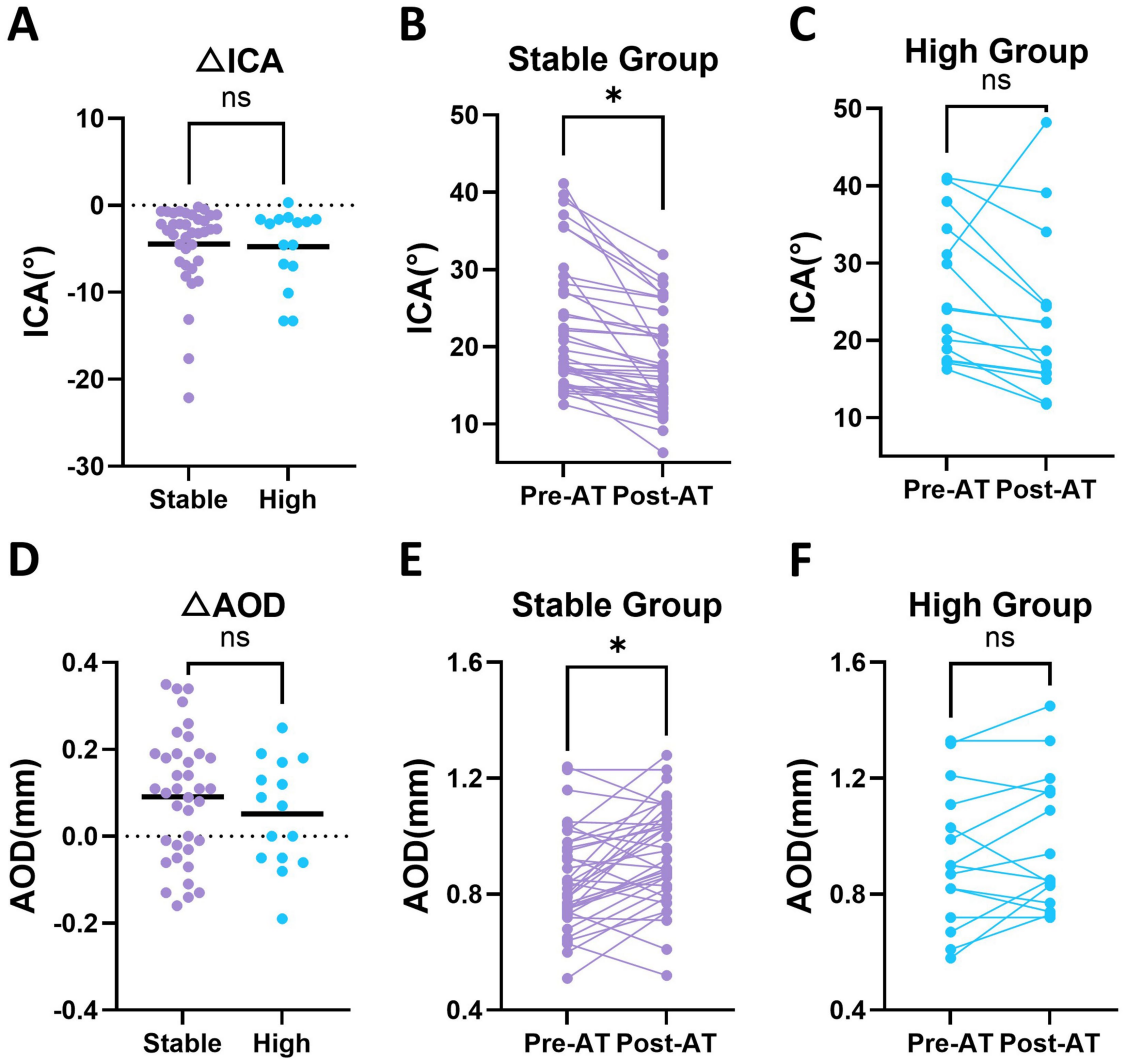
Baseline and post-atropine (post-AT) changes in iridocorneal angle (ICA) and angle-opening distance (AOD) in stable and high groups. **(A)** Change in ICA (ΔICA; post-AT minus pre-AT) in the stable and high groups. **(B,C)** Paired baseline and post-AT ICA values in the stable and high groups, respectively. **(D)** Change in AOD (ΔAOD; post-AT minus pre-AT) in the stable and high groups. **(E,F)** Paired baseline and post-AT AOD values in the stable and high groups, respectively. Statistical analyses were performed using linear mixed-effects models. Each dot represents an individual eye. Horizontal bars indicate mean values in panels **(A,D)**. Asterisks indicate statistically significant differences. Asterisks above paired data represent within-group comparisons between pre-AT and post-AT values, whereas asterisks above grouped data represent between-group comparisons. *p < 0.05, **p < 0.01, ***p < 0.001; ns, not significant.

Changes in anterior chamber parameters, including ICA and AOD, were analyzed using the LMM (Table 2). ICA showed a significant main effect of atropine administration (*p* < 0.01), reflecting a reduction at post-AT. This change was primarily observed in the stable group (22.32 ± 7.41 vs. 17.87 ± 7.41°, *p* = 0.012), whereas no significant change was detected in the high group (25.37 ± 8.39 vs. 20.60 ± 8.39°, *p* = 0.131). The group × atropine interaction was not significant (*p* = 0.926), indicating that the overall magnitude of ICA change did not differ significantly between groups (Figures 4A–C).

In contrast, AOD did not show a significant main effect of atropine administration (*p* = 0.089), and no significant group × atropine interaction was detected (*p* = 0.633). However, within-group comparisons revealed a significant increase in the stable group (0.85 ± 0.17 vs. 0.94 ± 0.17 mm, *p* = 0.025), whereas no significant change was observed in the high group (*p* = 0.558) (Figures 4D–F).

#### 3.4.2 CC changes

**Figure 5:**
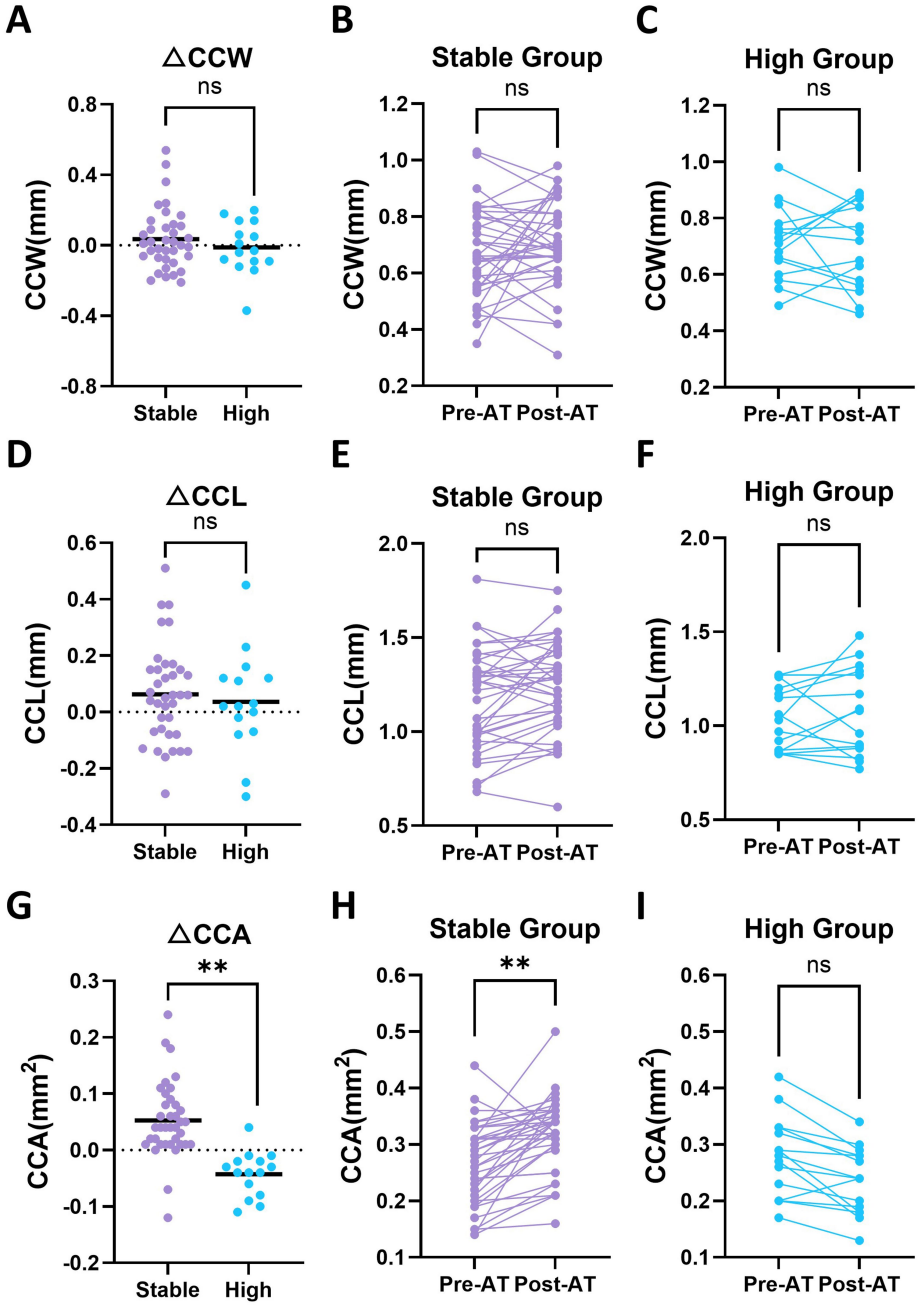
Baseline and post-atropine (post-AT) changes in ciliary cleft morphology in stable and high groups. **(A)** Change in ciliary cleft width (ΔCCW; post-AT minus pre-AT) in the stable and high groups. **(B,C)** Paired baseline and post-AT CCW values in the stable and high groups, respectively. **(D)** Change in ciliary cleft length (ΔCCL; post-AT minus pre-AT) in the stable and high groups. **(E,F)** Paired baseline and post-AT CCL values in the stable and high groups, respectively. **(G)** Change in ciliary cleft area (ΔCCA; post-AT minus pre-AT) in the stable and high groups. **(H,I)** Paired baseline and post-AT CCA values in the stable and high groups, respectively. Statistical analyses were performed using linear mixed-effects models. Each dot represents an individual eye. Horizontal bars indicate mean values in panels **(A,D,G)**. Asterisks indicate statistically significant differences. Asterisks above paired data represent within-group comparisons between pre-AT and post-AT values, whereas asterisks above grouped data represent between-group comparisons. *p < 0.05, **p < 0.01, ***p < 0.001; ns, not significant.

CC morphology was assessed using CCW, CCL, and CCA (Table 2). No significant group × atropine interaction was detected for CCW (*p* = 0.477) or CCL (*p* = 0.799), indicating that the magnitude of change in these parameters did not differ between the stable and high groups. Within-group comparisons also showed no significant changes in either group for CCW or CCL (Figures 5A–F). In contrast, a significant group × atropine interaction was observed for CCA (*p* = 0.002). Within-group comparisons revealed that CCA increased significantly in the stable group (0.27 ± 0.07 vs. 0.32 ± 0.07 mm^2^, *p* = 0.002), whereas a decreasing trend was observed in the high group (0.28 ± 0.07 vs. 0.24 ± 0.07 mm^2^, *p* = 0.084) (Figures 5G–I). These findings indicate distinct patterns of CCA change between groups at post-AT.

#### 3.4.3 Ciliary body changes

**Figure 6:**
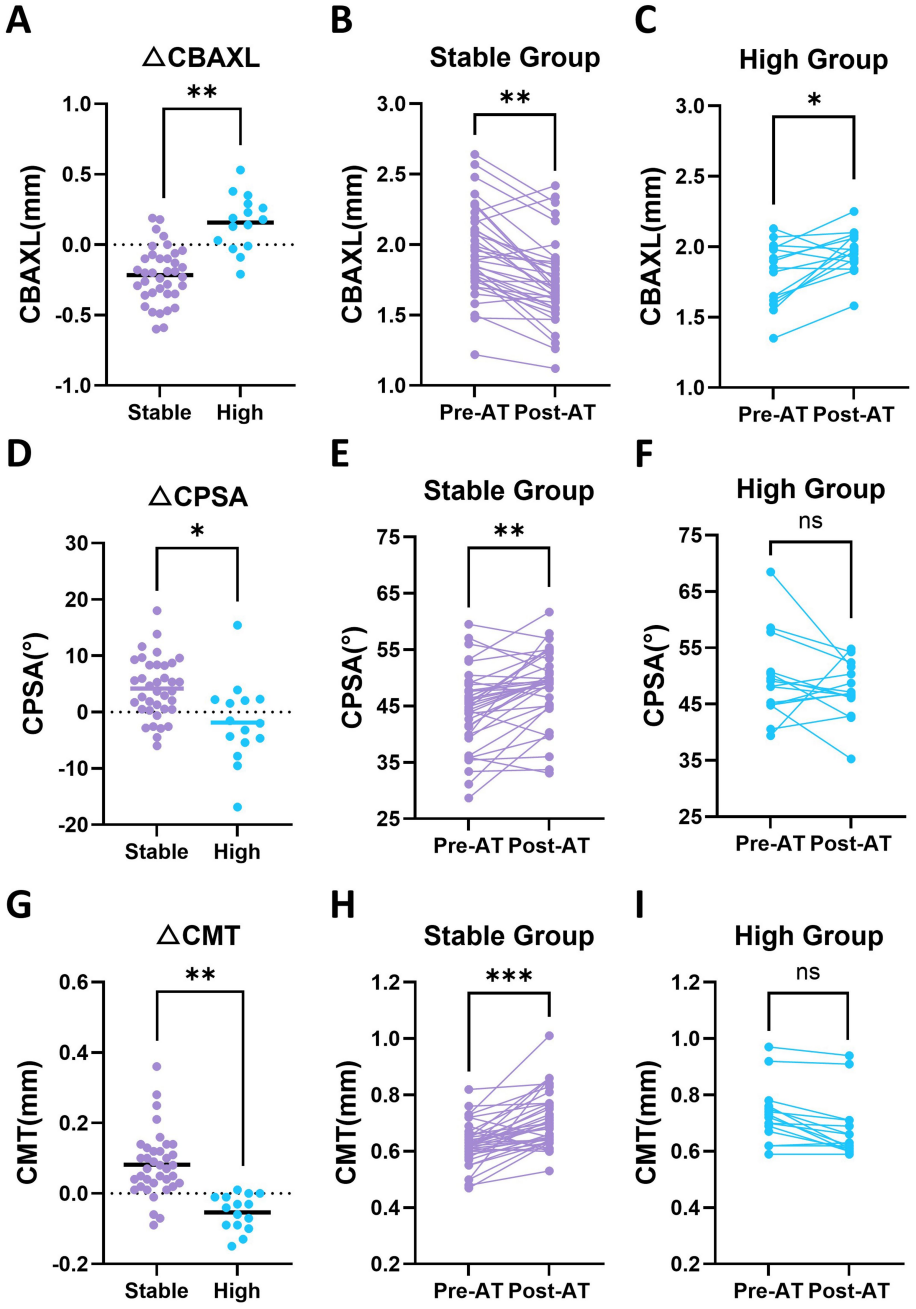
Baseline and post-atropine (post-AT) changes in ciliary body configuration in stable and high groups. **(A)** Change in ciliary body axial length (ΔCBAXL; post-AT minus pre-AT) in the stable and high groups. **(B,C)** Paired baseline and post-AT CBAXL values in the stable and high groups, respectively. **(D)** Change in ciliary process-sclera angle (ΔCPSA; post-AT minus pre-AT) in the stable and high groups. **(E,F)** Paired baseline and post-AT CPSA values in the stable and high groups, respectively. **(G)** Change in ciliary muscle thickness (ΔCMT; post-AT minus pre-AT) in the stable and high groups. **(H,I)** Paired baseline and post-AT CMT values in the stable and high groups, respectively. Statistical analyses were performed using linear mixed-effects models. Each dot represents an individual eye. Horizontal bars indicate mean values in panels **(A,D,G)**. Asterisks indicate statistically significant differences. Asterisks above paired data represent within-group comparisons between pre-AT and post-AT values, whereas asterisks above grouped data represent between-group comparisons. *p < 0.05, **p < 0.01, ***p < 0.001; ns, not significant.

Changes in ciliary body configuration were evaluated using CBAXL, CPSA, and CMT (Table 2). CBAXL showed a significant group × atropine interaction (*p* = 0.002), indicating opposite patterns of change between the stable and high groups. Within-group comparisons revealed a significant decrease in the stable group (1.95 ± 0.30 vs. 1.73 ± 0.30 mm, *p* = 0.003) and a significant increase in the high group (1.81 ± 0.19 vs. 1.96 ± 0.19 mm, *p* = 0.033) (Figures 6A–C). CPSA also showed a significant group × atropine interaction (*p* = 0.038). Within-group comparisons indicated a significant increase in the stable group (44.39 ± 6.69 vs. 48.58 ± 6.69°, *p* = 0.009), whereas no significant change was observed in the high group (*p* = 0.434) (Figures 6D–F). CMT did not show a significant main effect of atropine administration (*p* = 0.483); however, a significant group × atropine interaction was observed (*p* = 0.001). Within-group comparisons showed a significant increase in the stable group (0.63 ± 0.09 vs. 0.71 ± 0.09 mm, *p* < 0.001), whereas no significant change was observed in the high group (*p* = 0.177) (Figures 6G–I).

#### 3.4.4 Integrated summary of anterior segment changes

**Figure 7:**
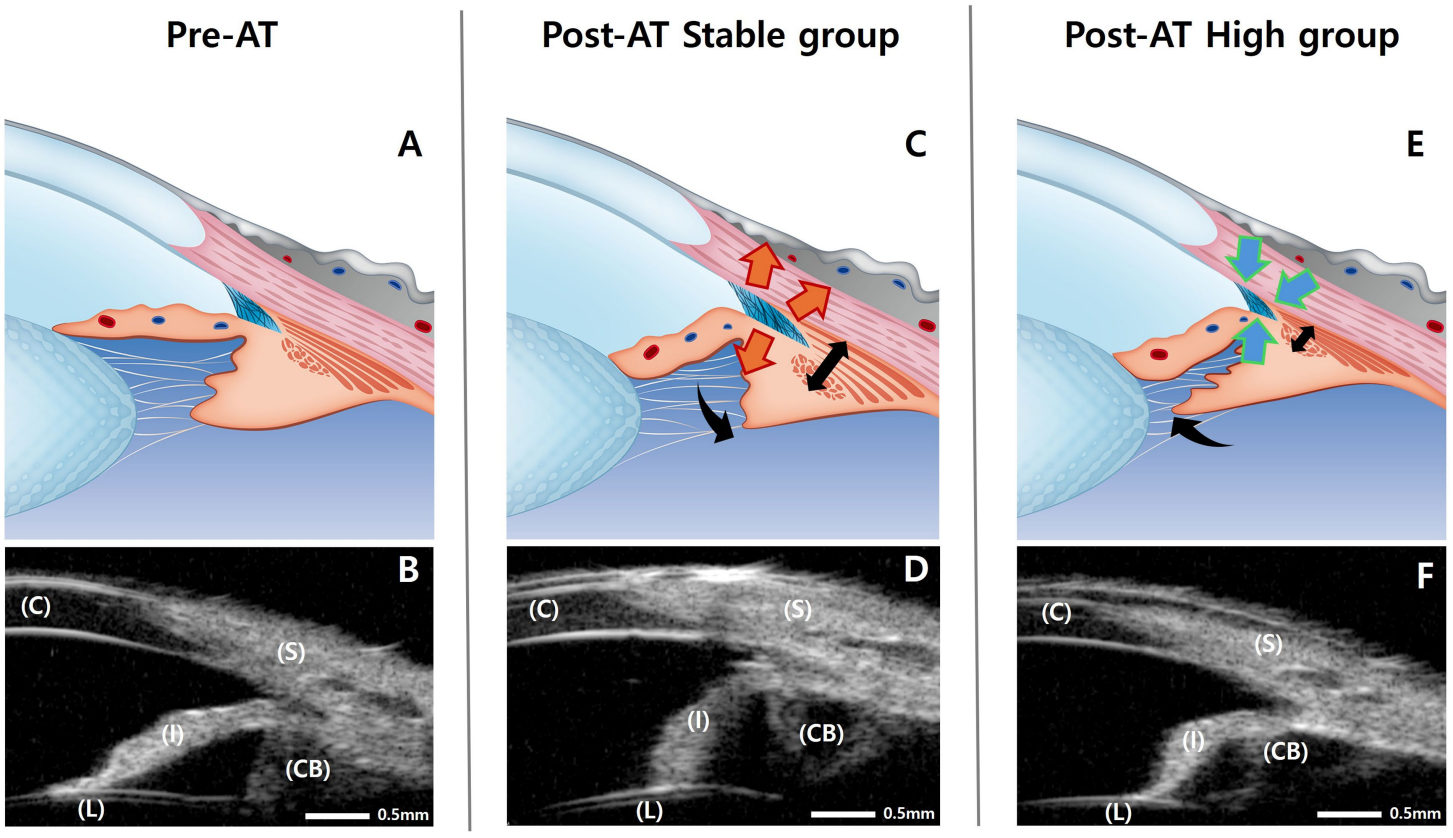
Schematic illustrations and corresponding ultrasound biomicroscopy (UBM) images showing anterior segment configurations before (Pre-AT) and after topical atropine administration (Post-AT). **(A,B)** Pre-AT (baseline) configuration of the anterior segment, shown schematically **(A)** and by UBM **(B)**. **(C,D)** Post-AT configuration in the stable group, illustrating posterior-outward displacement of the ciliary body with relative expansion of the ciliary cleft area (CCA) in the schematic **(C)** and corresponding UBM image **(D)**. **(E,F)** Post-AT configuration in the high group, illustrating anterior-inward displacement of the ciliary body with relative narrowing of the CCA in the schematic **(E)** and corresponding UBM image **(F)**. Arrows indicate the direction of apparent ciliary body and ciliary muscle displacement in the schematic representations. These illustrations summarize group-specific structural patterns observed at post-AT. Scale bars = 0.5 mm. CB, ciliary body; C, cornea; I, iris; L, lens; S, sclera.

When considered as a whole, anterior segment structural changes at post-AT differed between the response groups. Eyes in the stable group exhibited expansion of the CC (increased CCA), accompanied by posterior-outward displacement of the ciliary body (decreased CBAXL and increased CPSA). In contrast, eyes in the high group demonstrated narrowing of the CC (decreased CCA) and a tendency toward anterior-inward displacement of the ciliary body (increased CBAXL), with minimal change in CPSA. CMT showed a different pattern. In the stable group, CMT increased significantly at post-AT, whereas in the high group, CMT did not change significantly, although a decreasing trend was observed. Figure 7 provides a schematic summary of the structural differences between groups in relation to their IOP responses.

### 3.5 Correlation between anterior segment structural changes and IOP

**Figure 8:**
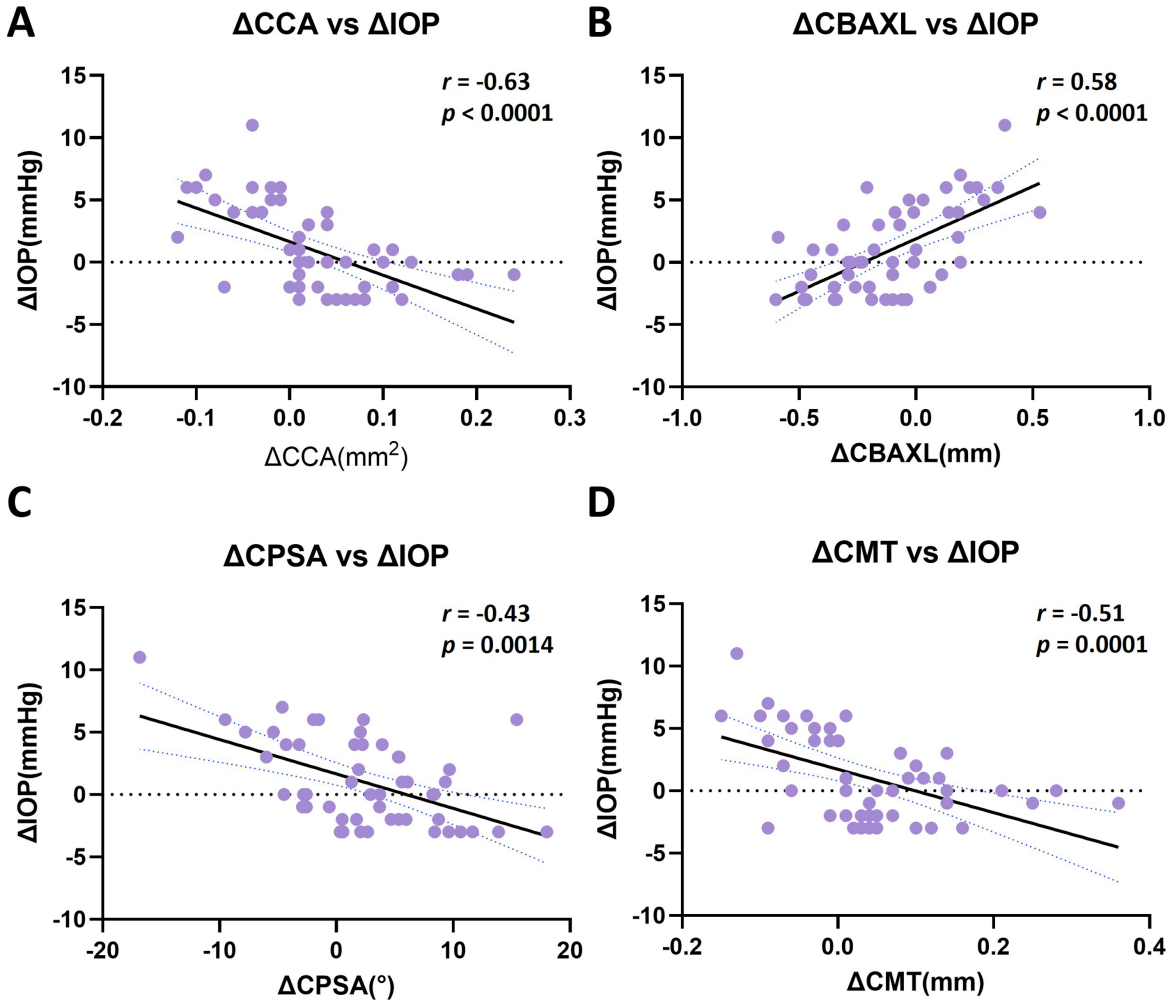
Correlation between changes in anterior segment parameters and intraocular pressure (IOP) following topical atropine administration. **(A–D)** Scatter plots showing the relationships between ΔIOP and changes in anterior segment parameters, including ΔCCA **(A)**, ΔCBAXL **(B)**, ΔCPSA **(C)**, and ΔCMT **(D)**. Each point represents an individual eye. Solid lines represent linear regression fits, and dotted lines indicate 95% confidence intervals. Significant correlations were observed between ΔIOP and each parameter shown. ΔCCA showed a significant negative correlation with ΔIOP, whereas ΔCBAXL showed a significant positive correlation. ΔCPSA and ΔCMT also demonstrated significant negative correlations with ΔIOP. Spearman correlation coefficients (*r*) and corresponding *p*-values are indicated in each panel.

Correlation analyses were performed to evaluate the relationships between changes in anterior segment parameters and ΔIOP at post-AT (Figure 8). Across all eyes, ΔIOP showed significant correlations with each parameter analyzed. ΔCCA demonstrated a significant negative correlation with ΔIOP (*r* = −0.63, *p* < 0.0001), whereas ΔCBAXL showed a significant positive correlation (*r* = 0.58, *p* < 0.0001). ΔCPSA and ΔCMT also exhibited significant negative correlations with ΔIOP (ΔCPSA: *r* = −0.43, *p* = 0.0014; ΔCMT: *r* = −0.51, *p* = 0.0001). Subgroup analyses were further performed within the stable and high groups (Supplementary Figures S1, S2). Correlations between structural changes and ΔIOP were inconsistent within individual groups. For ΔCMT, a significant negative correlation was identified only in the high group (*r* = −0.56, *p* = 0.032).

## 4 Discussion

IOP responses varied among eyes at post-AT. Based on this variation, eyes were classified into stable and high groups. Group-dependent differences appeared to be associated with distinct patterns of ciliary body motion and CC configuration.

In the present study, anterior segment structures were assessed using UBM, with a focus on their relationship to IOP responses. IOP reflects the balance between aqueous humor production and outflow, and evaluating pressure changes alongside structural alterations may help clarify the physiological effects of pharmacologic mydriasis (34). Variability in IOP responses after dilation suggests that some eyes may be more susceptible to pressure fluctuations, which may be related to underlying anatomical factors (35, 36).

Responses to pharmacologic dilation vary among eyes. Clinical studies in humans have described post-dilation IOP elevation in association with anterior chamber angle narrowing, iris displacement, and ciliary body changes (37, 38). In dogs, UBM-based studies have identified comparable structural changes, with CC narrowing observed after tropicamide administration and iridociliary alterations reported with 0.5% tropicamide–0.5% phenylephrine (TP) (5, 33). Although these studies offered structural insights, most analyses were conducted at the group level (4, 5, 24, 39). Eyes were typically pooled without considering whether IOP increased substantially or remained stable. This makes it difficult to interpret structural changes in the context of individual pressure responses. In our study, we therefore examined IOP and anterior segment morphology concurrently, with emphasis on identifying structural features that may underlie inter-individual variability in post-dilation IOP changes.

Human glaucoma and anterior segment imaging studies provide a useful comparative framework for interpreting these findings. In humans, pharmacologic dilation has been associated with angle narrowing and, in some cases, angle closure, particularly in anatomically predisposed eyes (38). Imaging studies using UBM and anterior segment OCT have also shown that ciliary body position can influence anterior segment configuration and aqueous humor outflow pathways (40, 41). Although direct comparison between species should be made with caution, these reports support the concept that structural variability following pharmacologic dilation may contribute to heterogeneous IOP responses. In this context, the group-dependent differences in ciliary body displacement and CC configuration observed in the present study may provide anatomical context for variable post-AT IOP responses in dogs.

Given the importance of evaluating both IOP and anterior segment structural changes, measurements were obtained at 20 min post-AT. This time point was selected based on previous evidence indicating that atropine-induced IOP elevation in dogs typically occurs shortly after administration and approaches its maximal response within approximately 20 min (20). Previous studies examining topical mydriatics in dogs have reported that IOP tends to increase within approximately 10 min post-AT, with peak elevations occurring around 20 min. The average increase relative to baseline was 2.6 ± 2.8 mmHg (24). Assessment at this interval permits evaluation of anterior segment morphology during the phase of near-maximal IOP response.

Canine IOP exhibits diurnal fluctuation, and changes within the physiologic range can influence clinical assessment (42). In the present study, a 4 mmHg threshold was used as a clinically oriented exploratory cutoff to identify eyes showing IOP increases exceeding the range typically observed in clinically normal dogs (25). Mean IOP increased slightly at post-AT (+0.9 ± 3.5 mmHg) and did not reach statistical significance, consistent with previous reports indicating average post-AT IOP changes of approximately 2–3 mmHg in normal dogs (20, 24). However, a subset of eyes showed more pronounced elevations, with 15 of 52 eyes (29%) demonstrating increases of ≥4 mmHg and a maximum increase of 11 mmHg, while the remaining eyes showed stable values or mild reductions. These findings support the use of this threshold as a clinically oriented method for identifying eyes with relatively greater post-AT IOP responses.

Importantly, this cutoff is consistent with our previous UBM-based study evaluating TP-induced IOP responses, allowing comparison of structural changes across different pharmacologic mydriatic agents (33). However, this threshold is best interpreted as an exploratory classification rather than a universally validated definition of responder eyes. To further evaluate the robustness of this classification, exploratory analyses using alternative thresholds (3 mmHg and 5 mmHg) showed similar overall patterns of group-dependent structural changes, particularly for parameters related to CC configuration and ciliary body position. These findings suggest that the observed associations are unlikely to be driven solely by the specific cutoff value and may reflect a consistent pattern of heterogeneous IOP response to atropine.

Interestingly, in a subset of dogs, one eye was classified as stable while the contralateral eye was classified as high. This intra-animal asymmetry suggests that factors beyond systemic or individual-level influences may contribute to variability in IOP response. Such differences may be related to subtle anatomical variations between eyes, including differences in ciliary body position, CC configuration, or local outflow resistance. In addition, aqueous humor outflow is known to be segmental rather than uniformly distributed, which may further contribute to asymmetric responses between eyes (13, 43–45). Variability in local pharmacologic response to atropine may also play a role (46). Although both eyes are exposed to the same systemic conditions, small differences in tissue responsiveness or baseline structural configuration may result in divergent IOP responses (46). These findings highlight the importance of considering eye-specific anatomical and functional factors when interpreting post-AT IOP changes.

At post-AT, ICA showed a mild reduction across all examined eyes. LMM analysis showed a significant main effect of atropine on ICA, with no significant group × atropine interaction. Although atropine significantly affected ICA, the absence of a group × atropine interaction indicates that the magnitude of angle narrowing did not differ between the stable and high groups. AOD did not show a significant change at post-AT, and no significant group × atropine interaction was observed. These findings indicate that atropine-related changes in traditional anterior chamber angle parameters occurred in a generally similar manner, irrespective of the subsequent IOP response. Despite mild ICA narrowing in the stable group, no corresponding increase in IOP was detected. Angle configuration changes alone do not appear to explain the IOP elevation observed at post-AT.

ICA and AOD measurements differ widely between dogs. These variations are more consistent with normal anatomical differences and technical factors than with true outflow dysfunction (5, 12). By contrast, IOP responses differed between groups and were accompanied by opposite changes in CC configuration. In the stable group, ICA decreased whereas CCA expanded. These concurrent changes may have offset the mild angle narrowing and contributed to stable IOP (28). Conversely, eyes in the high group showed an increase in IOP despite little change in ICA, suggesting that changes in CC configuration may be more closely associated with post-AT IOP elevation than traditional angle configuration alone (5, 28).

Previous experimental and imaging studies in humans and non-human primates have described characteristic changes in ciliary body configuration during accommodation, including anterior-inward displacement of the ciliary body accompanied by thinning of the ciliary muscle during contraction. These changes are commonly reflected by increases in CBAXL together with reductions in the CPSA, indicating an anterior shift in ciliary body orientation (29, 30). However, the applicability of this accommodation-based framework to canine eyes is limited by marked species-specific anatomical differences. In dogs, the ciliary muscle is less developed than in humans, and composite measurements of CMT may only indirectly represent muscle configuration. Consequently, CMT should be interpreted as a supportive parameter rather than a primary indicator of aqueous outflow dynamics (12, 47). Within this context, the group-dependent changes observed in the present study, namely opposing alterations in CBAXL and CPSA, are best interpreted as indicators of differential ciliary body displacement, rather than as direct analogs of accommodative contraction described in primate models.

In the present study, group-dependent patterns of ciliary body movement were observed following atropine-induced mydriasis. In the stable group, CBAXL decreased significantly and CPSA increased significantly, consistent with posterior-outward displacement of the ciliary body. This pattern was accompanied by a significant increase in composite CMT. In contrast, the high group showed a significant increase in CBAXL, whereas CPSA did not change significantly, indicating a relative anterior-inward shift of the ciliary body. In this group, CMT did not change significantly, although a decreasing trend was observed. These findings suggest that CMT changes reflect overall ciliary body configuration rather than direct muscle activity, as significant changes were observed only in the stable group. These opposing patterns indicate that atropine administration is associated with distinct ciliary body positional responses between groups, which may linked to differences in IOP behavior. However, as this was an observational study, these findings should be interpreted as associations rather than direct causal relationships.

Changes in CC morphology were consistent with the group-dependent patterns observed in ciliary body displacement. For CCL, values were lower in the high group than in the stable group, while no significant change related to atropine administration was observed. The CCA showed divergent patterns between groups, increasing significantly in the stable group. In contrast, the high group showed a decreasing trend in CCA, but this change did not reach statistical significance within the group. These findings indicate that CC morphology differed according to group-specific structural responses at post-AT. Such variations in CC configuration appear to be related to differences in ciliary body position. Nonetheless, species-specific anatomical characteristics should be considered when interpreting these findings. In humans and non-human primates, contraction of the ciliary muscle typically facilitates trabecular outflow by stretching the corneoscleral meshwork, whereas pupillary dilation may impede outflow by anterior displacement of the iris root (8, 48, 49). In dogs, however, contraction of the ciliary muscle is associated with anterior-inward movement of the ciliary body, which compresses the CC and is accompanied by increased resistance to aqueous outflow (28, 31, 33). Because the trabecular meshwork is located within the CC rather than anterior to the iris root, iris movement is thought to have a comparatively smaller influence on aqueous drainage in canine eyes. Accordingly, ciliary body displacement may play a more prominent role than iris configuration in regulating aqueous humor outflow in dogs (5, 12, 28).

Correlation analysis provided additional support for the association between structural alterations and IOP responses. In the overall dataset, ΔCCA showed a significant negative correlation with ΔIOP, indicating that narrowing of the CC was associated with IOP elevation, whereas expansion was associated with stable or decreased IOP. ΔCBAXL showed a positive correlation with ΔIOP, whereas ΔCPSA demonstrated a significant negative correlation. Together, these findings are consistent with an anterior-inward displacement of the ciliary body associated with increased IOP. However, these relationships were not consistently observed within individual response groups. This suggests that while structural changes are associated with IOP variation at the population level, the relationship may not follow a simple linear pattern within each group. Despite significant correlations observed in the overall dataset, no clear threshold or cut-off value was identified at which structural changes consistently resulted in IOP elevation. These findings indicate that atropine-induced IOP responses are influenced by a combination of structural factors rather than a single defining parameter.

Anatomically, the CC represents a shared outflow-related region for the trabecular and uveoscleral pathways in dogs (10–12). It includes trabecular structures associated with the trabecular pathway and is also contiguous with the ciliary body and supraciliary region involved in uveoscleral outflow (10–12). Therefore, the CC may function as a common structural component through which aqueous humor accesses both outflow routes. Accordingly, changes in CCA should not be interpreted as selective evidence of only one pathway, but rather as reflecting a shared outflow-related structural configuration. In the present study, enlargement of the CCA was associated with maintenance or reduction of IOP, whereas narrowing of the CCA coincided with IOP elevation. These findings suggest that changes in CCA reflect alterations in the configuration and accessibility of aqueous humor entry into outflow pathways, rather than a direct measure of outflow facility. Importantly, because aqueous humor dynamics were not directly measured, the present results should be interpreted as anatomical associations rather than pathway-specific mechanistic evidence.

In dogs, aqueous humor exits the eye primarily through two pathways: the trabecular outflow route and the uveoscleral pathway, with the latter accounting for approximately 15% of total aqueous drainage under normal conditions (50). Along the uveoscleral route, aqueous humor traverses inter-fiber spaces within the ciliary muscle, and resistance along this pathway is influenced by ciliary muscle tone (12). Relaxation of the ciliary muscle is associated with widening of these inter-fiber spaces, whereas muscle contraction reduces their caliber and increases resistance to outflow (12, 51). Pharmacologic studies have shown that atropine-induced relaxation can increase the relative contribution of the uveoscleral pathway, whereas pilocarpine exerts the opposite effect (52). In the stable group, the increase in composite CMT observed at post-AT is consistent with the overall pattern of ciliary body displacement and may reflect changes in ciliary muscle configuration. Although uveoscleral outflow was not directly measured in this study, such structural changes may be associated with reduced outflow resistance and stabilization of IOP.

Compared with a previous study using TP, the present atropine study demonstrated broadly similar overall structural patterns. In both studies, group-dependent differences in ciliary body-related parameters and CC configuration were associated with heterogeneous IOP responses (33). However, important differences in the pattern of ciliary body responses were observed between the two studies. In the TP study, eyes in the high IOP response group did not exhibit clear anterior displacement of the ciliary body but rather showed limited or absent relaxation compared to the stable group (33). In contrast, in the present atropine study, eyes in the high IOP response group demonstrated a tendency toward anterior displacement and a contraction-like configuration of the ciliary body. This suggests that, while both agents induce heterogeneous responses, the nature of that heterogeneity may differ. The TP-induced IOP elevation appears to be associated primarily with insufficient ciliary body relaxation, whereas atropine-induced IOP elevation may involve a more pronounced deviation toward anterior displacement in a subset of eyes. In line with these observations, the proportion of eyes classified into the high IOP response group was lower in the present atropine study (29%) compared with the TP study (37%).

This difference may reflect a greater tendency for atropine to induce posterior-outward displacement of the ciliary body in most eyes, while a smaller subset exhibits an opposing response pattern (15, 31). Although alterations in CMT were observed, their relationship to the direction of ciliary body displacement remains uncertain. It is conceivable that differences in ciliary muscle configuration may contribute to the heterogeneous movement patterns, including anterior displacement observed in a subset of eyes. However, given the technical limitations of UBM in accurately characterizing ciliary muscle contraction states, this interpretation should be approached with caution and warrants further investigation. Although direct comparisons of aqueous humor dynamics between studies were not performed, these findings suggest that drug-specific differences in the direction and magnitude of ciliary body movement, rather than changes in traditional anterior chamber angle parameters, may be an important determinant of variability in post-mydriatic IOP responses.

This study has several limitations. All measurements were obtained at a single time point, approximately 20 min post-AT (20, 24). Previous studies have shown that atropine-induced IOP changes in dogs generally approach a maximal response within this interval. This time point allowed assessment of anterior segment morphology near the expected early peak of IOP response. The use of a single observation window provides only a limited view of atropine-related effects. The full temporal profile cannot be fully characterized within this time frame. The cycloplegic effect of atropine can last up to 2 weeks in dogs (53). Related structural or IOP changes may therefore extend beyond the time frame evaluated in this study. Variation in the timing and magnitude of mydriatic and cycloplegic responses between eyes may lead to additional structural changes, such as further ciliary body relaxation or positional shifts after this time point in some eyes. The temporal relationship between iris dilation and ciliary body movement may also differ between eyes, potentially contributing to variability in later structural responses and IOP changes. The associated IOP response may persist beyond the evaluated time point and may also change over time. Evaluating multiple post-AT time points in future studies may help to better characterize these structural and IOP changes.

A formal acclimatization period prior to IOP measurement was not included. This may have contributed to variability related to stress or handling, although examinations were conducted in a calm environment to minimize such effects. It should also be noted that most of the dogs included in this study were small-breed dogs, which may limit generalizability to larger breeds. Differences in ocular anatomy and anterior segment configuration between small- and large-breed dogs could influence aqueous humor dynamics and associated structural responses (26, 54). In this study, UBM measurements were limited to the dorsal quadrant. Aqueous humor outflow is known to be segmental rather than uniformly distributed, as demonstrated in human and experimental studies (55–57). Although direct evidence in dogs remains limited, similar regional variability may be present and could contribute to variability in anterior segment measurements obtained from a single quadrant. Evaluating multiple quadrants or the entire circumference in future studies may help to better characterize these changes. Interobserver variability was also not assessed in this study. All measurements were performed by a single examiner, ensuring consistency in measurement technique. However, this approach limits assessment of interobserver reproducibility.

Several methodological limitations should also be considered when interpreting CMT measurements. Evaluating CMT using UBM in dogs is technically challenging. The ciliary muscle changes shape during both relaxation and contraction, and image resolution limits clear identification of muscle boundaries. As a result, composite CMT measurements primarily reflect overall muscle configuration. Discrete changes at the fiber level are less likely to be captured. Measurement consistency may be influenced by anatomical differences among dogs, as well as variability in the visualization of scleral reference points across imaging systems (12, 31).

In summary, group-dependent patterns of ciliary body displacement and CC configuration were associated with heterogeneous post-AT IOP responses in dogs. Specifically, eyes in the high group showed anterior displacement of the ciliary body, with a tendency toward inward rotation, together with a tendency toward narrowing of the CCA, whereas eyes in the stable group exhibited posterior-outward displacement accompanied by CCA expansion. These structural patterns were associated with corresponding differences in IOP responses, and changes in CCA may reflect anatomical configurations relevant to this variability. Changes in composite CMT provided supportive information regarding overall ciliary body configuration but should be interpreted cautiously given methodological limitations. Assessment of ciliary body position and CC morphology may therefore provide useful structural context for interpreting atropine-induced IOP changes. Overall, these findings provide structural context for atropine-induced IOP variation and should be interpreted as anatomical associations rather than pathway-specific mechanistic evidence.

## Data Availability

The raw data supporting the conclusions of this article will be made available by the authors, without undue reservation.
