# Supplementary material for: Atropine-induced intraocular pressure changes and anterior segment structural parameters in dogs assessed by ultrasound biomicroscopy
Source: Front Vet Sci. 2026 May 20;13:1820977. doi: 10.3389/fvets.2026.1820977 (PMC13229754; doi:10.3389/fvets.2026.1820977)

**Figure S1. Subgroup correlation analyses between changes in ciliary cleft area (CCA), ciliary body axial length (CBAXL), and intraocular pressure (IOP) in stable and high groups.**

(A, B) Correlations between ΔCCA and ΔIOP in the stable (A) and high (B) groups. (C, D) Correlations between ΔCBAXL and ΔIOP in the stable (C) and high (D) groups. Each point represents an individual eye. Solid lines represent linear regression fits, and dotted lines indicate 95% confidence intervals. Spearman correlation coefficients (r) and corresponding *p*-values are shown in each panel. No significant correlations were observed within either group.

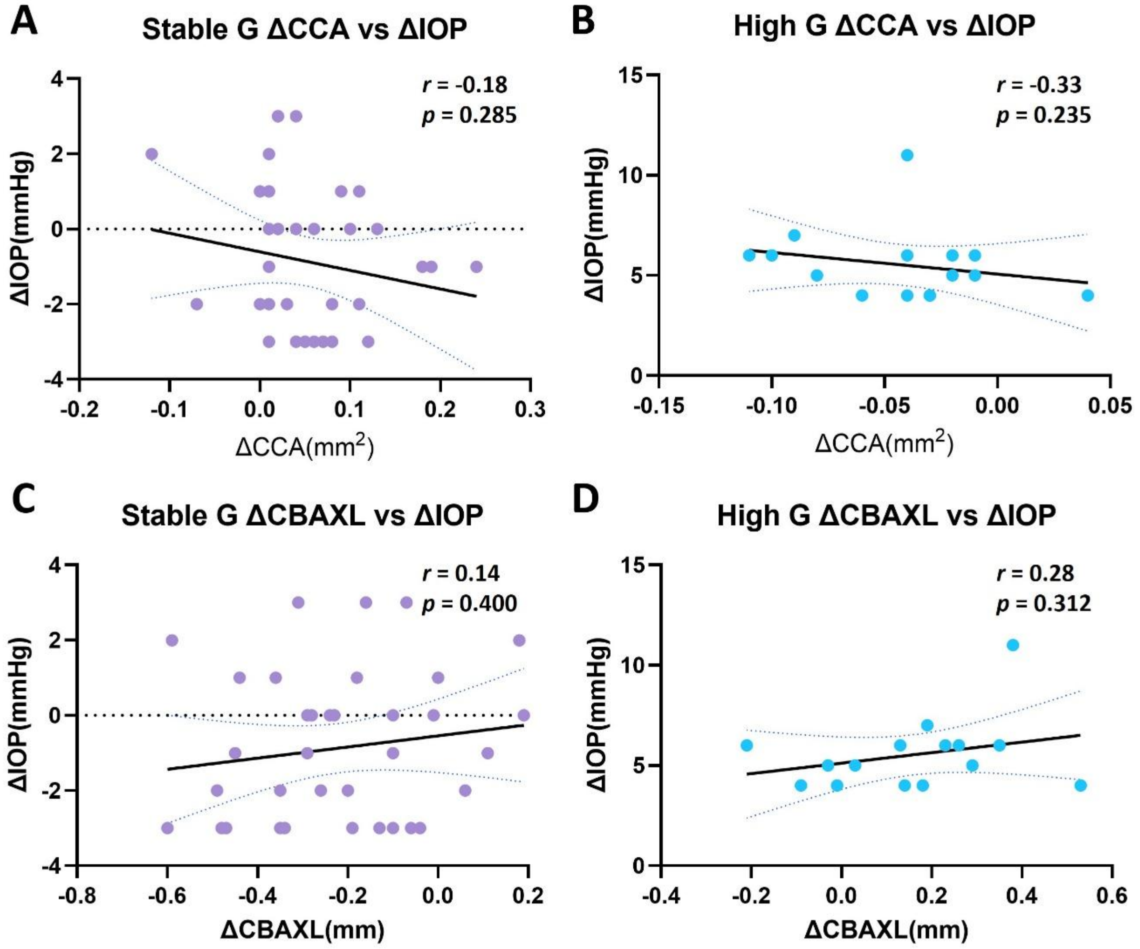

**Figure S2. Subgroup correlation analyses between changes in ciliary process-sclera angle (CPSA), ciliary muscle thickness (CMT), and intraocular pressure (IOP) in stable and high groups.**

(A, B) Correlations between ΔCPSA and ΔIOP in the stable (A) and high (B) groups. (C, D) Correlations between ΔCMT and ΔIOP in the stable (C) and high (D) groups. Each point represents an individual eye. Solid lines represent linear regression fits, and dotted lines indicate 95% confidence intervals. Spearman correlation coefficients (r) and corresponding *p*-values are shown in each panel. No significant correlations were observed for ΔCPSA in either group. For ΔCMT, no significant correlation was observed in the stable group, whereas a significant negative correlation was observed in the high group.

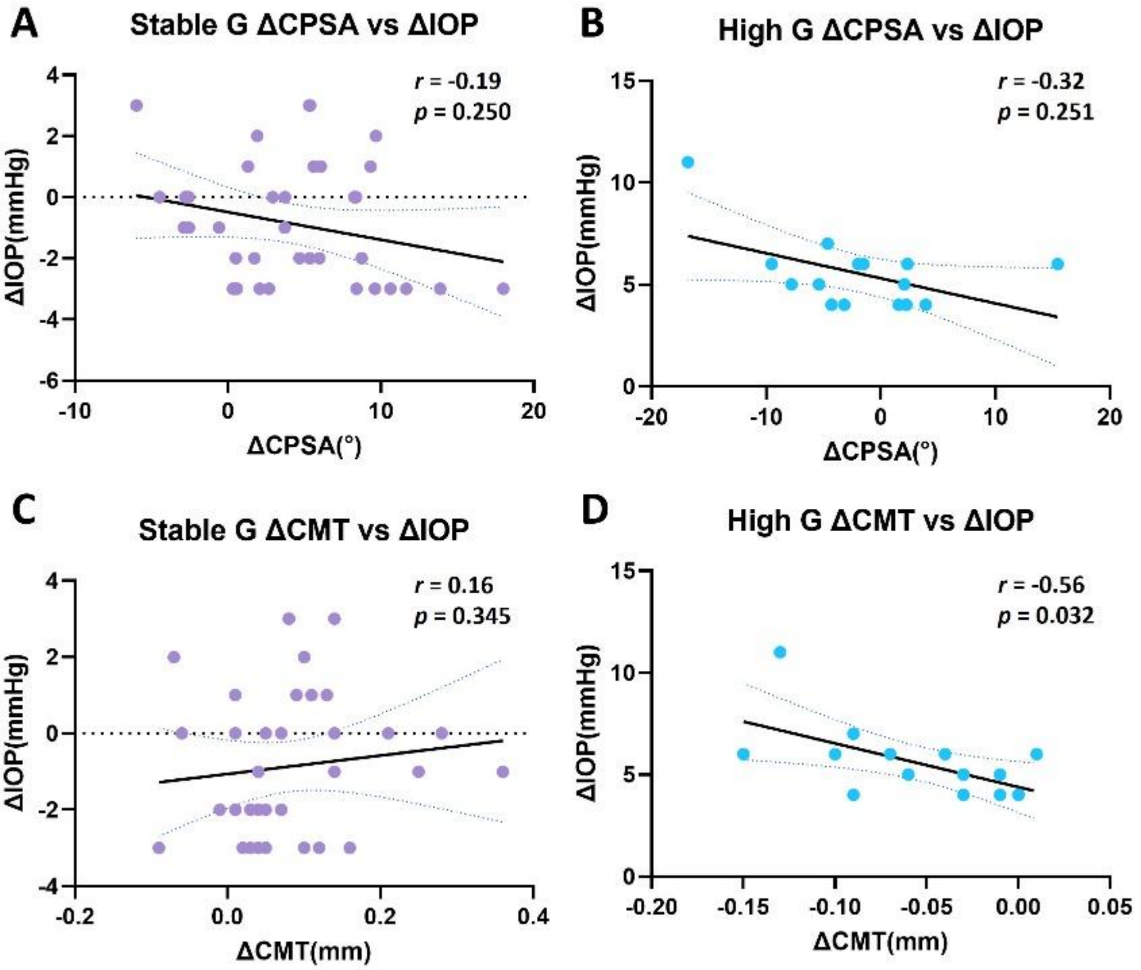

Supplement: Supplementary file 1 [file Image_1.pdf]
